# Factors Associated with Testing and Prompt Use of Recommended Antimalarials following Malaria Diagnosis: A Secondary Analysis of 2011-12 Tanzania HIV and Malaria Indicator Survey Data

**DOI:** 10.1371/journal.pone.0132964

**Published:** 2015-07-17

**Authors:** Juma Adinan, Damian J. Damian, Sia E. Msuya

**Affiliations:** 1 Institute of Public Health, Department of Epidemiology and Biostatistics, Kilimanjaro Christian Medical University College (KCMU Co), Moshi, Tanzania; 2 Institute of Public Health, Department of Community Health, Kilimanjaro Christian Medical University College (KCMU Co), Moshi, Tanzania; 3 Department of Community Health, Kilimanjaro Christian Medical Centre (KCMC) Hospital, Moshi, Tanzania; 4 Assistant Medical Officer (AMO)-General Teaching College, KCMC Hospital, Moshi, Tanzania; Johns Hopkins University, UNITED STATES

## Abstract

**Introduction:**

Malaria is still a public health problem in Sub-Saharan Africa. Malaria causes mortality mostly in children under-five years. Early detection and prompt treatment using recommended antimalarials is key to malaria control. However, in Tanzania, contrary to the national goals, a large proportion of children with fever taken to health facilities are not tested for malaria and those tested positive are not promptly treated using recommended antimalarials. The aim of this study was to determine factors associated with malaria testing and prompt use of recommended antimalarials among under-five children with fever in Tanzania.

**Methods:**

This was a secondary analysis of Tanzania HIV and Malaria Indicator Survey (THMIS) data 2011-12 obtained from a national cross sectional survey. The analysis involved children aged 6-59 months whose mothers reported they had fever two weeks preceding the survey. Factors associated with testing and uses of recommended antimalarials were obtained using logistic regression.

**Results:**

Of the 1675 under-five children with fever, 951 (56.8%) were taken to the health facilities. Of the 951 children, only 394 (41.48%) febrile children were tested for malaria. Of those tested, 291 (78.91%) were diagnosed with malaria. Of those diagnosed with malaria, only 124 (42.68%) children used recommended antimalarials within 1st 24 hours of diagnosis. In multivariate analysis, children taken to health centers (OR 1.79; 95%CI: 1.07 - 3.00) and to the hospitals (OR 3.4; 95%CI: 1.75 - 6.77) had higher odds of being tested compared to those taken to dispensary and other lower level health facilities. Children were more likely to use recommended antimalarial promptly if they had a caretaker with secondary or higher education (OR: 4.07; 95%CI: 0.61 - 2.68) or living in the rural area (OR: 3.21; 95%CI: 1.09 - 9.44) compared to those with an uneducated caretaker or from an urban area.

**Conclusion:**

Training on malaria testing and treatment guidelines should be provided, and preventing stock outs of malaria testing kits and medications at dispensary level should be made available as it is the first point of health care for most Tanzanians. Reasons on why urban people are less likely to use recommended antimalarials need to be investigated and addressed for proper malaria management.

## Introduction

Malaria is still a public health problem especially in SSA [1]. It kills mostly children under-five years. In Tanzania malaria is the second cause of under-five morbidity and mortality, contributing to 10% of under-five mortality [2]. Malaria symptoms overlap with other microbial and viral diseases. However, fever, in malaria endemic areas is the key symptom to offer malaria treatment [3]. The WHO malaria treatment guideline recommends that parasitological confirmation should be done on all suspected malaria cases. Those found positive, should be treated promptly (within 24 hours of diagnosis) using recommended antimalarials [4]. It is therefore important for caretakers of children with fever to take their children to health facilities where malaria testing can be done. Malaria is a preventable and treatable disease, however if not timely diagnosed and treated uncomplicated malaria progresses to severe malaria which often leads to death [5].

In Tanzania, approximately 90% of the population lives within five kilometers of a health facility [6]. However, only 40–54% of caretakers seek healthcare at health facilities when their children have fever [3]. Malaria Rapid Diagnostic Tests (mRDT) have been shown to have high sensitivity and specificity in detecting malaria parasites in malaria endemic areas [7–9]. Universal use of mRDT at facilities has been shown to improve rational use of antimalarials and reduce development of resistance and so recommended for scaling-up [8, 10].

Despite the introduction of mRDT in malaria diagnosis in 2011, in Tanzania, contrary to the national goals [11], large proportion of children with fever who are taken to health facilities are not tested for malaria [12] and those who test positive are not promptly treated using recommended antimalarials [13]. Furthermore, there are documentations on the use of recommended antimalarials without malaria testing [14–15]. This phenomena of failure to test for malaria among children presenting at health facilities with fever and treating febrile children who might not have malaria with antimalarials, leads to the mismanagement of the patient resulting into missed opportunity for timely treatment of children with malaria and misuse of antimalarials for those clinically diagnosed with malaria who might, in fact, have bacterial or viral illness and are negative for malaria [16–17].

While studies have assessed factors influencing testing for malaria among children; facility factors [18], clinician factors [19] and patients’ social cultural factors [20], little work has been done on the factors associated with using recommended antimalarials within the first 24 hours after diagnosis of positive malaria test. The few studies that addressed the use of antimalarials reported on the use of antimalarials among febrile children and not those diagnosed with malaria [3, 21, 22].

This study analyzed the Tanzania HIV and Malaria Indicator Survey (THMIS) of 2011–12, which is the most recent in a series of country surveys, with the following objectives; 1) to determine factors associated with malaria testing and 2) to determine factors associated with prompt use of recommended antimalarials following malaria diagnosis among under-five year children taken to health facilities by their caretakers.

## Methodology

This is a secondary analysis of data from Tanzania HIV and Malaria indicator survey (THMIS) 2011–2012 which is the third and most recent in a series of national sample surveys that aims to estimate the key indicators of malaria and HIV for each Tanzania’s region.

THMIS is a nationally-representative cross-sectional household surveys that are performed in an interval of four years. The information can be generalized from the region, zonal and national level, and not below these levels. Sample was obtained by using two stage probability sampling, first stage involved selection of clusters and second stage selection of households. Clusters were selected from a list of enumeration areas of the 2002 Population and Housing Census. In the 2011–12 THMIS 583 clusters were selected from which 10,496 households participated in the survey. In total, 8648 mothers with under-five children were interviewed.

In individual questionnaire, female respondents were asked questions to collect information on several topics including but not limited to; socio-demographic characteristics, sexual and reproductive history, and on knowledge of malaria and HIV. Information about recent fever and treatment of fever for children who were less than five years old was also collected during interviews as well as place of care and treatment received. The Household Questionnaire also collected information on characteristics of the household to determine the wealth index of households, ownership of mosquito nets and recording biomarkers test results, anemia for children under-five years old [22].

The data set which was used is that with children’s information i.e. Children Records (KR dataset). The unit of analysis in this study was children under five (5) years old (age 6–59 months) and their mothers.

### Recording of some important variables

Testing for malaria variable was obtained based on the reported information from the caretaker that children with fever two weeks preceding the survey were taken to the health facility, had blood taken either from a heel or a finger. Prompt use of recommended antimalarials- this variable was created by labeling those who used recommended antimalarials within 24 hours of diagnosis, as shown in the Tanzanian Malaria Diagnosis and Treatment guidelines. Children who were positive for malaria but used antimalarial after twenty four hours were labeled otherwise. Wealth index- this is a re-coding of the wealth index variable present in the dataset in three categories contrary to the five categories present in the dataset. This variable was created by compressing the original wealth index into tertile. Instead of poorest, poor, middle, rich, richest the newly re-coded wealth index was put into poor, middle and rich categories.

### Data analysis

STATA Version 12 (Stata Corp., Texas, and US.) was used in data processing and analysis accounting for the complex sampling design of the THMIS, clustering effect. Descriptive analysis included computations of frequency and percentages for categorical variables and measures of central tendency and respective measures of dispersion for continuous variables. Using Logistic Regression, crude and adjusted Odds Ratios were obtained to assess the strength of association between independent variables and the outcome variables. P-value of less than (5%) with 95% Confidence interval were used to measure statistical significance of the association between independent and dependent variables.

Denominator for analyzing factors associated with testing for malaria were determined by analyzing children who were reported to have fever two weeks preceding the survey and whose mothers sought care to health facility. The denominator for prompt use of recommended antimalarials were obtained by analyzing children who were reported to have fever two weeks preceding the survey, whose mothers sought care to health facility and tested positive for malaria.

### Ethical consideration

During data collection, the broad goals of the exercise were explained to the respondents by fieldworkers during their introduction in the household and written consent was obtained from all participants prior to all the data collection procedures [22]. The ethical clearance to conduct this study was obtained from Kilimanjaro Christian Medical University College (KCMU-Co), Kilimanjaro, Tanzania. Participants’ records were anonymized and de-identified by DHS-Program before being released to the public domain. Permission to use this data was obtained from the DHS PROGRAM.

## Results

### Baseline characteristics of children

Of the 8573 children studied, 1675 had fever two weeks preceding the survey. Of these 1675, 51% were males. The median age of children included in this study was 23 (IQR; 14–38) months. The majority of the 1675 children were residing in rural 1359 (81.1%) and from families with low wealth index 790 (47.2%), Table 1.

**Table 1:** Social demographic characteristics of under five children with fever two weeks preceding the survey (n = 1675).

Variables	N	%
**Sex**		
*Male*	854	51.00
*Female*	821	49.00
**Age in months**		
*06–11*	256	16.60
*12–23*	509	33.10
*24–59*	774	50.30
**Residence**		
*Urban*	316	18.90
*Rural*	1359	81.10
***Education level of mother***		
No formal education	372	22.20
Primary	1157	69.10
Secondary and more	146	08.70
***Marital status of the mother/caregiver*?**		
Never	97	05.80
Married	1395	83.20
Separated	184	11.00
***Employment status of the mother/caregiver*?**		
No	1501	89.70
Yes	173	10.30
***Age in years of the mother/caregiver***		
15–25	615	36.80
26–35	725	43.30
36–49	335	20.00

### Malaria testing and treatment

**Fig 1:**
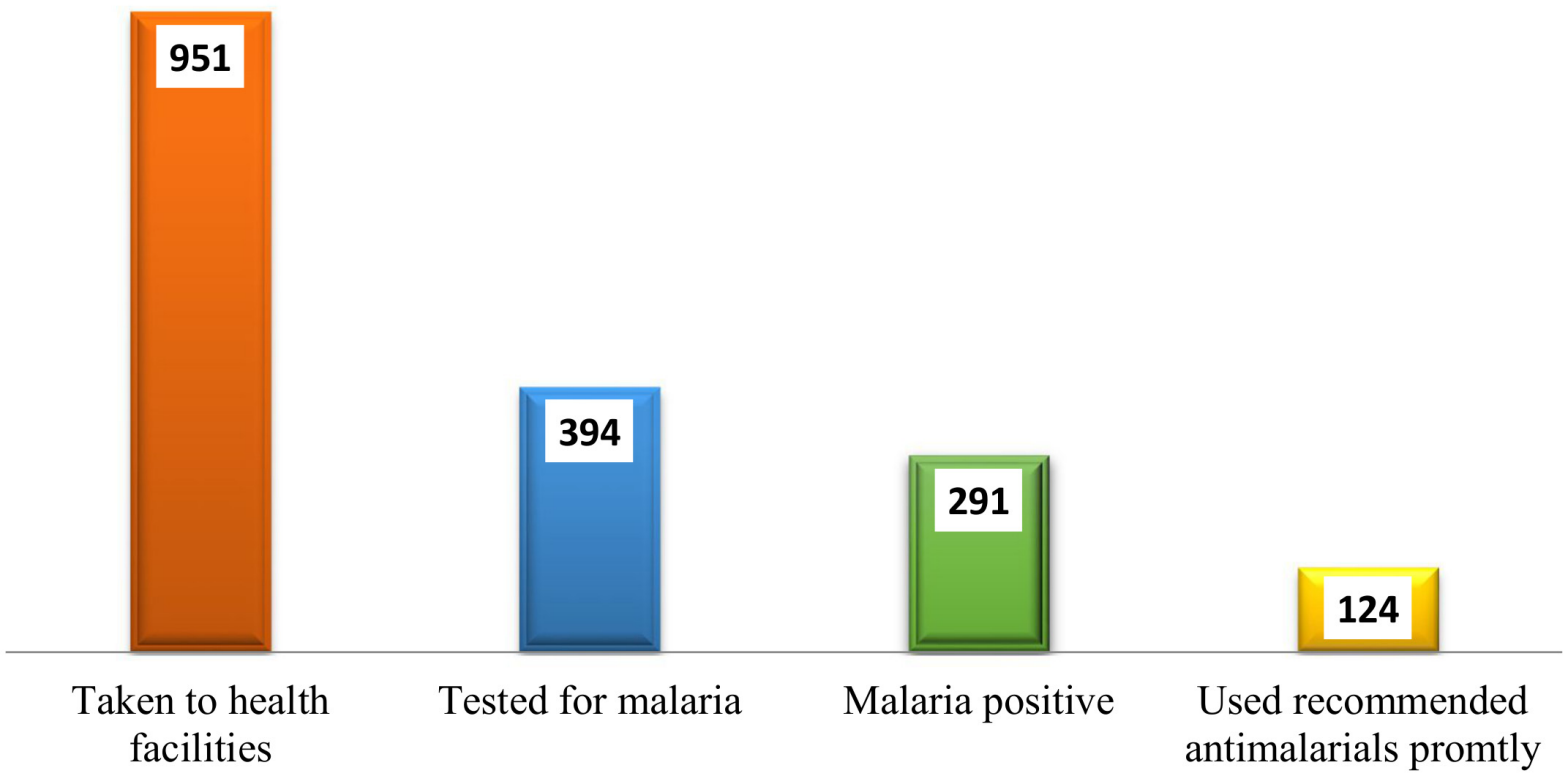
Number of febrile children who sought appropriate health care, got tested and used recommended antimalarials promptly.

Of the 1675 febrile children, 1312 (78.3%) were given care or taken for health care at pharmacies, health facility or treated at the household level. In this analysis health facilities are taken to be appropriate place for care seeking of children with fever because malaria suspected cases can be checked by mRDT and can be appropriately managed. Nine hundred and fifty one children (56.76%) were taken to appropriate health facilities (dispensary, health center and hospital), had appropriate healthcare seeking behavior (AHCSB). Of the 951 febrile children whose mothers sought appropriate healthcare, 394 (41.48%) were tested for malaria. Of the 394 febrile children tested, 291 (78.91%) tested positive for malaria. Of the 291 malaria positive children, 266 (91.35%) children were treated using any antimalarials. Two hundred and nine (71.72%) children were treated using recommended antimalarials (which was artemether-lumafantrine ALu) any time after being diagnosed. Of the 291 children diagnosed with malaria, only 124 (42.68%) children used recommended antimalarials promptly i.e. the same or next day after being diagnosed with malaria as shown in Fig 1.

Of the 124 children who used recommended antimalarials, 74 (59.68%) used Artememther Lumefantrine, followed by Quinine 44 (35.48%), three children (2.42%) diagnosed with malaria used Artesunate and Amodiaquine and 3 (2.42%) used Artesunate. Substantial number of malaria cases 33 (11.34%) got treated using regimen not recommended on the guideline by 2011 (Amodiaquine alone 26, Sulfadoxine Pyrimethamine 2). Others, 5 (1.72%) used antimalarials which were not specified while 25 (8.59%) others used nothing after being diagnosed with malaria.

Of the 556 (58.46%) children with fever who were taken to appropriate healthcare facilities but not tested, 305 (32.07%) got treated using any antimalarials anytime while of these, 167 (17.56%) used recommended antimalarials promptly.

### Factors associated with malaria testing among under-five children with fever taken to appropriate healthcare facilities

Children living in rural areas have 79% less odds of being tested compared to those in urban areas (OR 0.21; 95%CI: 0.08–0.24). Also, children taken to health centers and hospitals have high odds of being tested compared to those taken to dispensary and other lower levels. The odds of testing are 1.8 (OR 1.79; 95%CI: 1.07–3.00) and 3.4 (OR 3.4; 95%CI: 1.75–6.77) times higher for children taken to health centers or hospitals respectively compared to those taken to dispensary and other lower level health facilities, Table 2.

**Table 2:** Factors associated with malaria testing among under-five children with fever taken to appropriate healthcare facilities (n = 951). Significant at *** p<0.001; ** p<0.01; and * p< 0.05. Ref. Reference category. Adjusted for all variables.

	AHCSB‡ (N = 951)	TEST(N = 394)		Unadjusted Analysis		Adjusted Analysis	
Variables	Freq	Freq	%	OR	95%CI	OR	95%CI
***Child sex***							
Male	509	219	43.20	Ref.		Ref.	
Female	442	175	39.50	0.86	0.64–1.16	1.07	0.76–1.52
***Child Age (months)***							
06–11	170	69	40.40	Ref.		Ref.	
12–23	284	117	41.30	0.86	0.55–1.35	0.89	0.55–1.45
24–59	413	182	44.10	0.89	0.60–1.32	0.81	0.53–1.25
Missing	84	26					
***Residence***							
Urban	235	178	75.60	Ref.		Ref.	
Rural	716	216	30.30	0.14***	0.08–0.24	0.21***	0.08–0.53
***Education***							
No formal education	173	60	34.70	Ref.		Ref.	
Primary	662	263	39.80	1.24	0.73–2.11	0.74	0.42–1.30
Secondary and more	116	71	61.40	2.98**	1.53–5.82	1.05	0.46–2.36
***Marital status***							
Never	73	28	39.10	Ref.		Ref.	
Married	777	324	41.70	1.12	0.64–1.95	2.40	0.99–5.80
Separated	101	42	41.20	1.09	0.47–2.52	2.75	0.96–7.89
***Employment Status***							
Yes	826	315	38.20	Ref.		Ref.	
No	125	79	63.30	2.79***	1.55–5.03	1.22	0.62–2.40
***Wealth index***							
Low	385	107	27.90	1		1	
Middle	344	125	36.30	1.47	0.95–2.27	1.34	0.86–2.10
High	222	162	73.10	7.02***	4.10–12.02	1.85	0.74–4.61
***Age of the mothers***							
15–25	364	155	42.60	1.51	0.99–2.32	1.49	0.93–2.41
26–35	413	182	44.10	1.61	0.99–2.60	1.22	0.74–2.01
36–49	174	57	32.90	Ref.		Ref.	
***Type of Health Facility***							
Hospital	131	91	70.20	4.62***	2.75–7.77	3.44***	1.75–6.77
Health Center	144	75	51.80	2.11***	1.36–3.28	1.79*	1.07–3.00
Dispensary	676	228	33.70	Ref.		Ref.	

^‡^AHCSB = appropriate health care seeking behavior

### Factors associated with prompt use of recommended antimalarials (PURA)

Urban residence, having not-working caretaker and caretaker with secondary or higher education have been shown to be associated with prompt use of recommended antimalarials following malaria diagnosis (P<0.05).

Children living in rural have three times higher odds of using recommended antimalarials promptly following malaria diagnosis compared to their urban counterparts (OR: 3.21; 95%CI: 1.09–9.44) while, children with caretaker who have secondary education and more have four times higher odds of using recommended antimalarials promptly following malaria diagnosis compared to those with caretaker with no education (OR: 4.07; 95%CI: 0.61–2.68). For children with not-working caretaker, the odds of using recommended antimalarials promptly following a diagnosis of malaria is four times higher compared to those children with working caretaker (OR: 4.04; 95%CI: 1.31–12.41), Table 3.

**Table 3:** Factors associated with prompt use of recommended antimalarials following malaria diagnosis n = 291. Significant at * p< 0.05. Ref. Reference category. Adjusted for all variables.

	PURA‡			Unadjusted Analysis		Adjusted Analysis	
Variables	n = 291	Frequency	%	OR	95%CI	OR	95%CI
***Child sex***							
Male	168	68	40.80	Ref.		Ref.	
Female	123	56	45.20	1.20	0.69–2.08	1.26	0.68–2.34
***Child Age (months)***							
06–11	52	20	37.60	0.87	0.41–1.85	0.51	0.23–1.10
12–23	77	42	54.70	1.74	0.87–3.48	1.55	0.75–3.21
24–59	139	57	41.00	Ref.		Ref.	
Missing	23	5					
***Residence***							
Urban	142	56	39.50	Ref.		Ref.	
Rural	149	68	45.70	1.29	0.73–2.30	3.21*	1.09–9.44
***Education***							
No formal education	41	19	46.90	Ref.		Ref.	
Primary	198	76	38.20	0.70	0.29–1.69	0.94	0.38–2.32
Secondary and more	53	29	56.20	1.45	0.48–4.37	4.07*	1.13–14.64
***Marital status***							
Never	24	8	34.20	Ref.		Ref.	
Married	237	105	44.30	1.53	0.54–4.40	2.28	0.61–8.53
Separated	31	11	36.80	1.12	0.28–4.45	1.65	0.33–8.38
***Employment Status***							
Yes	238	97	41.00	Ref.		Ref.	
No	54	27	50.20	1.45	0.69–3.03	4.04*	1.31–12.41
***Wealth index***							
Low	73	31	42.40	Ref.		Ref.	
Middle	88	40	45.60	1.14	0.58–2.27	1.23	0.57–2.68
High	130	53	40.90	0.94	0.47–1.90	1.23	0.41–3.68
***Age of the mothers***							
15–25	105	41	38.90	1.12	0.51–2.47	1.17	0.48–2.86
26–35	143	68	47.40	1.58	0.64–3.90	1.43	0.57–3.64
36–49	43	15	36.30	Ref.		Ref.	
***Type of Health Facility***							
Hospital	68	29	41.80	0.94	0.46–1.89	1.22	0.62–2.41
Health Center	50	20	41.40	0.92	0.45–1.89	0.72	0.30–1.73
Dispensary	173	75	43.40	Ref.		Ref.	

^‡^PUR = prompt use of recommended antimalarials

## Discussion

This study determined factors associated with testing and prompt use of recommended antimalarials. The results show that still there is substantial number of febrile children under-five years that are not tested for malaria despite being taken to the health facilities and very few of those diagnosed with malaria use recommended antimalarials and within recommended time.

Furthermore, the results showed factors associated with malaria testing are residence and type of the health facility febrile children were taken to while factors associated with prompt use of recommended antimalarials after being diagnosed with malaria are residence, working status of the caretaker and the education level of the caretaker.

Less than 50% of the children with fever taken to appropriate health facilities were tested for malaria. This proportion is very low compared to the Tanzanian guideline for managing malaria which requires testing to be universal for every child presenting with fever at the health facilities [23]. The low proportion of testing could be explained by the clinician practices [19, 24, 25, 26] or by frequent stock-out of diagnostic tests [26] as highlighted in the service availability and readiness survey [26] report. Malaria testing is an important element in increasing rational use of antimalarials prescription [7, 9, 27], however as shown, in this study, it is still a key bottleneck toward appropriate malaria management in Tanzania, and this requires urgent attention. Studies need to be conducted at facility levels to have a clear understanding of this situation.

One of the goal in Tanzania medium term malaria strategic plan is to have at least 80% of positive malaria cases receiving recommended antimalarials within 24 hours after being diagnosed with malaria [11]. But from the results of this study, the proportion of under-five children using recommended antimalarials with 24 hours following malaria diagnosis is very low (38.5%). These results are in line with that published by other researchers from different African countries including Tanzania [3, 13, 28, 29]. However, this finding contradicts result from Uganda where there was 90% use of recommended antimalarials following malaria diagnosis. This difference could be explained by the effect of close follow-up of trained healthcare workers on the use of malaria rapid diagnostic test [27]. Studies elsewhere have shown unavailability of recommended antimalarials [26] to be a key reason for failure to give antimalarials promptly. Despite seeking care at health facilities and timely diagnosis of malaria, the health care system misses the opportunity of treating children promptly with recommend antimalarials and this can result into progression to severe malaria and possibly avoidable deaths.

Further, more than 30% of the febrile children who were not tested for malaria used antimalarials. This finding is similar with the results reported in other parts of sub-Saharan Africa [18,24,30]. This, as it has been shown in other parts, may signify the reluctance of healthcare providers in adhering to new guidelines of managing malaria patients. Earlier, the management of malaria among children was mainly based on presenting symptoms and signs as directed in the IMCI guideline.. This finding and that of low usage of testing for children with fever who are brought to health facilities emphasize the need for proper training of health care providers on the guidelines as they change over time.

In this study, testing for malaria was done more at hospitals and health centers than at dispensaries. In Tanzania nearly 70% of the dispensaries are in rural areas where 80% of Tanzanians live, while most hospitals are in urban [26]. This means the majority of Tanzanian children do receive sub-standard care when they attend dispensaries with fever. Training of providers in malaria management, uninterrupted supply of malaria diagnostic kits and medications and close monitoring of the practices in malaria management should be strengthened and scaled-up at dispensary and rural areas of Tanzania.

The strength of this study lies on the fact that the data is representative of the Tanzania population which strengthens the generalisability of the results. Also mothers were asked on a history of fever and its management within two weeks preceding the survey which decreases recalling bias. The weakness point of this study is that it analyzed only individual variables collected by THMIS survey but failed to explore other factors which plausibly are associated with outcomes of interest, malaria testing and prompt use of recommended antimalarials, for example health facility factors. The second limitation to this study is it cross sectional nature which is always good to develop hypothesis but cannot tell the causal relationship between independent factors and outcomes.

### Conclusion

To effectively control malaria, low proportion of malaria testing and low proportion of prompt use of recommended antimalarials following malaria diagnosis should be addressed through frequent training on guidelines, provision of malaria testing devices and close monitoring. Further studies are needed on why children in urban areas are not given recommended antimalarial despite being more frequently tested for malaria.
